# Impact of surgical checklist and its completion on complications and mortality in urgent colorectal procedures

**DOI:** 10.1590/0100-6991e-20213031

**Published:** 2022-02-18

**Authors:** CAMILA SARMENTO GAMA, CHANTAL BACKMAN, ADRIANA CRISTINA OLIVEIRA

**Affiliations:** 1 - Prefeitura de Belo Horizonte, Secretaria Municipal de Saúde - Belo Horizonte - MG - Brasil; 2 - University of Ottawa, School of Nursing - Ottawa - Ontario - Canadá; 3 - Universidade Federal de Minas Gerais, Escola de Enfermagem - Belo Horizonte - MG - Brasil

**Keywords:** Patient Safety, Colorectal Surgery, Checklist, Emergencies, World Health Organization, Segurança do Paciente, Cirurgia Colorretal, Lista de Checagem, Emergências, Organização Mundial da Saúde

## Abstract

**Objective:**

to assess the impact of using a surgical checklist and its completion on complications such as surgical site infection (SSI), reoperation, readmission, and mortality in patients subjected to urgent colorectal procedures, as well as the reasons for non adherence to this instrument in this scenario, in a university hospital in Ottawa, Canada.

**Methods:**

this is a retrospective, epidemiological study. We collected data from an electronic database containing information on patients undergoing urgent colorectal operations, and analyzed the occurrence of SSI, reoperation, readmission, and death in a 30 day period, as well as the completion of the checklist. We conducted a descriptive statistical analysis and logistic regression.

**Results:**

we included 5,145 records, of which 5,083 (98.8%) had complete checklists. As for the outcomes evaluated, cases with complete checklists displayed higher SSI rate, 9.1% vs. 6.5% (p=0.466), lower reoperation rate, 5% vs.11.3% (p=0.023), lower readmission rates, 7.2% vs. 11.3% (p=0.209), and lower mortality, 3.0% vs. 6.5% (p=0.108) than cases with incomplete ones*.*

**Conclusion:**

there was a high level of checklist completion and a larger number of the outcomes in the reduced percentage of incomplete checklists found, demonstrating the impact of its utilization on the safety of patients undergoing urgent operations.

## RESUMO

**Objetivo:**

verificar o impacto do uso do *checklist* cirúrgico e a completude em complicações como infecção do sítio cirúrgico (ISC), reoperação, readmissão e mortalidade em pacientes submetidos a procedimentos colorretais de urgência, bem como os motivos para a não adesão a esse instrumento nesse cenário, em hospital universitário de Ottawa, Canadá.

**Métodos:**

trata-se de estudo epidemiológico retrospectivo. Os dados foram coletados em base de dados eletrônica contendo informações de pacientes submetidos a cirurgias colorretais de urgência, sendo analisada a ocorrência de ISC, reoperação, readmissão e óbito em período de 30 dias, bem como a completude do *checklist*. Realizou-se análise estatística descritiva e regressão logística.

**Resultados:**

incluíram-se 5.145 registros, dos quais 5.083 (98,8%) possuíam *checklists* completos. No que se refere aos desfechos avaliados, identificou-se nos checklists completos comparados aos incompletos, respectivamente, maior taxa das ISC de 9,1% contra 6,5% (p=0,466); menor taxa em reoperações de 5% contra 11,3% (p=0,023); em readmissões de 7,2% contra 11,3% (p=0,209); e também em mortalidade de 3,0% contra 6,5% (p=0,108).

**Conclusão:**

verificou-se alto nível de completude do *checklist* e maior número de desfechos no reduzido percentual de *checklists* incompletos encontrados, demonstrando o impacto da utilização para a segurança do paciente submetido a cirurgias de urgência.

## INTRODUCTION

In 2009, the World Health Organization (WHO) launched the Second Global Patient Security Challenge: “Safe Surgeries Save Lives”, aimed at the improvement of care to the surgical patient, to extend the safety and reduce the morbidity and mortality arising from surgical procedures. The use of a surgical checklist was proposed with the aim of assisting surgical teams at reminding critical steps to be performed during the procedures and at promoting the improvement of interdisciplinary communication^1^. 

The implementation of the checklist has caused the reduction of postoperative complications and mortality worldwide, especially in elective procedures^2^. In 2010, with the purpose of evaluating the use of the surgical checklist during urgent operations, the WHO conducted a study in different socioeconomic realities, whose results have shown decreased rates of complications and deaths after the introduction of the checklist, suggesting its viability in these circumstances^3^. 

Since then, few studies have focused on this scenario^4^ ^-^ ^6^. Despite the positive results on morbidity and mortality^3^ ^,^ ^6^ and the recommendation of the WHO^1^ for its implementation in urgent procedures, its use in these situations has been questioned by some healthcare workers, arguing that the application could lead to other problems such as delay in the procedure^7^. Although patients undergoing emergency operations may benefit from the use of the checklist, since they present greater risk of complications and mortality due to their instability, associated with the pressure of emergency care in a short space of time^3^, evidence are still scarce about the impact of the checklist completion in this population^3^ ^,^ ^4^ ^,^ ^6^. 

In an emergency procedures scenario, colorectal surgeries stand out, which are associated with high rates of morbidity and, consequently, higher demand for hospital resources. The postoperative complications arising from these procedures are surgical site infection (SSI), anastomotic leak, and intestinal obstruction that many times require intensive care, increasing admission time, reoperations, and readmission, as well as health expenditure^8^ ^-^ ^9^. 

In lieu of this, we aimed at verifying the impact of the use of the surgical checklist and its completion on complications such as SSI, reoperation, readmission, and mortality in patients undergoing urgent colorectal procedures, as well as the reasons for non-adherence to this instrument in this scenario in a university hospital at Ottawa, Canada. The present study aims to contribute with evidence on the relationship between the occurrence of adverse events (AE) - SSI, reoperation, readmission, and death - and the surgical checklist completion in urgent colorectal procedures, circumstances, many times, subject to the pressure of time and, therefore, failures.

## METHODS

We conducted a retrospective, epidemiological study in a large university hospital in Ottawa, Canada, certified by Accreditation Canada, in which the checklist was introduced in April 2010. 

The checklist adopted in the institution is composed of three phases. The first is “sign in”, consisting of verification of safety items, such as patient identification, procedure, surgical site and allergies, signature of the informed consent term, assessment of patient’s airways, risk of hypothermia, need of special precautions, instruments sterilization, operation of the anesthetics equipment, reserves of blood products, documentation of β HCG exam, equipment status, among others, when the patient arrives at the operative room. This phase involves all the perioperative staff, including, among others, the anesthesiologist, the surgeon, the medical assistants, the resident, the nurse, and the scrub nurse. It is recommended that this step be led by the surgeon or medical assistant and may be conducted by the resident in the presence of one of the first two, as well as in the next step, called “time out”. 

During “time out”, the time immediately after anesthetic induction and before surgical incision, one checks again the patient’s identity, the operation site and the signing of the procedure’s informed consent, the need and presence of imaging exams, thromboembolism prophylaxis, antibiotic prophylaxis, as well as the description of any concerns with potential complications by the attending surgeon, prior to the procedure. In the third phase, the “sign out”, which is held during the suture and before the patient’s transfer to the anesthetic recovery room, one should check the record of the performed procedure name, the operative wound classification, the count of surgical instruments and packs, the identification of specimens for the anatomopathology laboratory, and the occurrence of AE and intraoperative complications to be reported to the unit that will receive the patient. This last phase must be carried out by the room scrub nurse, in the presence of the surgeon, the medical assistant, and the anesthesiologist. It is emphasized that the scrub nurse is the responsible for electronically documenting the conclusion of each checklist phase and the reasons for any non-completion, if applicable. 

We collected the data retrospectively in the hospital’s database, comprising all patients older than 18 years, undergoing urgent colorectal surgery, such that the clinical conditions demanded access to the OR in up to 24 hours after admission, in the period of August 1^st^, 2010 and July 31^st^, 2017. We included information on SSI, reoperation, readmission, and death 30 days after the procedure, as well as sex, age, procedure performed, American Society of Anesthesiologist (ASA) score, surgical wound classification, procedure duration, and the checklist completion or the reasons for it not having been completed. 

We selected for the study colorectal procedures (operations in the appendix, colon, and rectum), such as laparoscopic appendectomy, exploratory laparotomy, resection/operation of the small bowel, hemicolectomy, and colectomy, due to the high volume held in the institution in the study period. We excluded records of patients with ASA 5 classification due to the decreased chance of survival without operation, ASA 6, since it means a patient in brain death for organ donation, or ASA score not recorded^10^. 

We analyzed the data by descriptive statistics, X^2^ test, and logistic regression to assess the impact of the checklist completion on SSI, reoperation, readmission, and death, with the software Statistical Package for the Social Sciences (SPSS), version 21.0. We considered a value of p < 0.05 as significant. To assess data normality, we used the Kolmogorov Smirnov test. We used the variables that characterized patients and procedures to control possible confounding factors. The study was approved by the Research Ethics Board of the institution (# 20170449-01H).

## RESULTS

We included 5,145 records in the analysis. Patients’ mean age was of 48.8 years (18-99) and the average duration of the operation was 135 minutes (60-584). We found that 5,083 (98.8%) checklists were complete in all three phases (sign in, time out, and sign out) and 62 (1.2%) were incomplete. 

 As for the incomplete checklists, the incomplete phases were “sign out” in 24 (38.7%), followed by “time out” in 15 (24.2%), “sign in” and “time out” in 14 (22.6%), “sign in” in six (9.7%), “time out” and “sign out” in two (3.2%), and “sign in” and “sign out” in one (1.6%). The most frequently reported reasons for checklist incompletion were “absence of a team member”, this member most frequently being the surgeon (76.9%), and “patient’s clinical instability”, as described in Table 1.

**Table 1:** Reasons for the non adherence to the different checklist phases. Ottawa, 2017.

Reason	N (%)
Absence of a team member	26 (41.9)
Patient’s clinical instability	17 (27.4)
Occupied / distracted	7 (11.3)
Forgetfulness	6 (9.7)
Non adherence to checklist specific items	5 (8.1)
Unidentified	1 (1, 6)
Total	62 (100)

The characteristics of patients and procedures according to checklist completion stratified by sex, age, ASA score, surgical wound, and procedure duration (calculated from sample mean) are described in Table 2.

**Table 2:** Characteristics of the procedure according to checklist completion. Ottawa, 2017.

Variables	Complete N (%)	Incomplete N (%)	X^2^	Total N = 5,145
Sex	N = 5. 083	N = 62	0.131	
Female	2,622 (99)	26 (1)		2,648 (51.5)
Male	2,461(98.6)	36 (1.4)		2,497 (48.5)
Age			0.802	
18 49 years	2,624 (98.8)	33 (1.2)		2,657 (51.6)
≥ 50 years	2,459 (98.8)	29 (1.2)		2,488 (48.4)
ASA			0.000	
II	2,392 (98.9)	26 (1.1)		2,418 (47.0)
III	1,525 (99.5)	8 (0.5)		1,533 (29.8)
IV	1,166 (97.7)	28 (2.3)		1,194 (23.2)
Variables	Complete N (%)	Incomplete N (%)	X^2^	Total N = 5,145
Surgical wound			0.952	
Potentially contaminated	4,414 (98.8)	54 (1.2)		4,468 (86.8)
Contaminated	669 (98.8)	8 (1.2)		677 (13.2)
Procedure duration			0.047	
60 135 minutes	3,321 (99)	33 (1)		3,354 (65.2)
≥ 136 minutes	1,762 (98.4)	29 (1.6)		1,791 (34.8)

**Figure 1:**
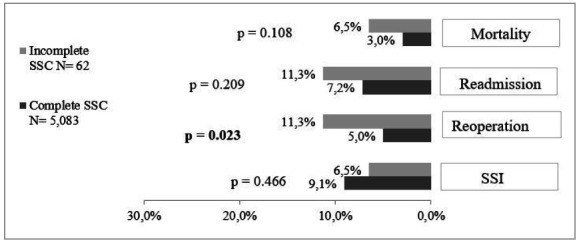
Distribution of clinical outcomes according to checklist completion. Ottawa, 2017.

Regarding the outcomes analyzed, we identified the occurrence of SSI in 9.1% (468/5,145), reoperations in 5% (259/5,145), readmission in 7.2% (370/5,145), and mortality of 3% (154/5,145). The distribution of outcomes according to checklist completion is described in Figure 1.

We carried out the logistic regression analysis while controlling for the possible confounding factors, including sex, age, ASA score, surgical wound classifications, procedure duration, and checklist completion, to assess the impact on SSI, reoperations, readmissions, and deaths. Checklist completion did not influence the occurrence of any of the evaluated outcomes (Table 3).

**Table 3:** Logistic regression analysis of outcomes evaluated according to patients’ characteristics, procedure, and checklist completion. Ottawa, 2017.

	Outcomes											
Variables	SSI			Reoperation		Readmission	Mortality			Mortalidade		
	p	OR	IC (95%)	p	OR	IC (95%)	p	OR	IC (95%)	p	OR	IC (95%)
Constant	0.000	0.009		0.000	0.006		0.000	0.026		0.978	0.002	
Checklist completion (reference= complete)	-	-	-	0.340	1.515	0.646 3.556	-	-	-	-	-	-
	Outcomes											
Variables	SSI			Reoperation		Readmission	Mortality			Mortalidade		
Age (reference= 18 49 years)	0.360	1.125	0.874 1.447	0.161	0.794	0.576 1.096	0.577	1.078	0.829 1.402	0.000	6.471	3,195 - 13,106
Gender (reference= female)	-	-	-	-	-	-	-	-	-	-	-	-
ASA classification (reference=II)	0.000			0.000			0.000			0.000	8.387	5,371-13,097
III	0.000	7.529	4.830 11.737	0.000	6.598	3.561 12.225	0.000	2.788	1.998 3.891	**	**	**
IV	0.000	15.737	10.018 24.721	0.000	33.687	18.404 61.660	0.000	3.643	2.542 5.221	**	**	**
Wound Classification (reference= potentially contaminated)	0.000	1.973	1.561 2.494	0.004	1.560	1.154 2.108	0.605	1.079	0.810 1.437	0 139	1.3 40	0,910-1,975
Duration of operation (reference= 60-135 minutes)	0.000	2.013	1.601 2.532	0.641	1.069	0.807 1.418	0.000	1.689	1.322 2.159	0.073	1.394	0,969-2,004

**In the regression analysis of the outcome “mortality”, it was necessary to group ASA II and III categories and consider them as reference for purposes of comparison with ASA 4. This strategy was used to present more consistent data, as there were no deaths in ASA 2 category.

## DISCUSSION

The high rate of checklist completion found in this study demonstrates the feasibility of its adopting in this scenario. As for the impact of completion in the evaluated outcomes, it is noteworthy that despite the reduced quantity of incomplete checklists (1.2%), in those there were higher rates of the outcomes reoperation, readmission, and death, the first being statistically significant. This result allows to infer that the proper use of the surgical checklist in urgent procedures is possible and promising and may reduce complications and mortality. Similarly, a study that evaluated the influence of the checklist completion in emergency procedures also identified positive effects in reducing the rates of complications and mortality. Although lower than those previously reported by other authors, this result was significantly related to the adhesion to the checklist^6^. On the other hand, it is appropriate to highlight the absence of impact of the surgical checklist completion on SSI. According to the literature, one possible explanation for this result would be the largest investment in developed countries in infection prevention and control policies^11^ ^,^ ^12^, the surgical checklist being an extra tool that, depending on the development / adaptation, may add little to the good practice measures already adopted in the workflow by health institutions in these scenarios^13^ ^,^ ^14^. 

Regarding the logistic regression, we could not identify any impact of the checklist completion on the assessed outcomes. A possible explanation for this result is based on the small number of incomplete checklists found, which is one of the limitations of the present study. Authors that evaluated the use of the checklist in urgent procedures with positive results in the evaluated outcomes reported a more homogeneous distribution of data between the periods evaluated, before and after the checklist implementation, as well as of complete and incomplete checklists^3^ ^,^ ^4^ ^,^ ^6^.

As for the reasons for the non filling of the checklist, we highlight the “absence of a team member”, mostly represented by the surgeon, as the most reported reason, surpassing “patient’s clinical instability”, the main reason for non adherence to the instrument in urgent procedures according to the literature^6^ ^,^ ^7^. This finding reflects the failure of teamwork and the presence of a hierarchical culture historically implicit in operating rooms all over the world, in which surgeons are the coordinators^15^ ^,^ ^16^. Some authors point out that when the leadership of the checklist is taken by this professional, other staff members tend to concentrate and to carry out the verifications together. In addition, they do it in less time and with greater information exchange than when the responsibility of conducting the checklist is taken by the nursing staff, as usually occurs^16^ ^,^ ^17^. 

 Appreciation and understanding of the magnitude of teamwork are fundamental to the checklist success. The positive results after its use found by the WHO and by other authors that included emergency surgical procedures in the analysis^3^ ^,^ ^4^ ^,^ ^6^ were influenced by the change in behavior of the surgical staff and extensive training^5^. With regards to training, some authors refer to the difficulty of implementing the checklist describing the lack of knowledge of professionals about the purpose and relevance of the process. Many times, this is seen as one more purposeless bureaucratic task devoid of practical usefulness, as a top-down directive to control the decision or even to reduce professional autonomy^16^ ^-^ ^19^. Kearns et al. stated that the surgical team was less prone to believe that the checklist would be inconvenient in urgent surgical procedures after getting accustomed to its utilization in elective cases, demonstrating the importance of educating the staff for its use with confidence. 

 Another aspect to be considered is local adaptation, which advocates the development of the checklist with the participation of an inter disciplinary staff, avoiding an excessive number of items to be checked, which can increase the probability of one specific item being disregarded^18^. In this sense, one alternative to the checklist adaptation to emergency situations, aimed at greater completion and adherence, is the reduction of the number of items that constitute it, since simplicity and applicability have been favorable factors to its adoption^1^ ^,^ ^3^. In the present study, the institution used one checklist containing 45 items in all procedures, elective or urgent, which may have contributed to the completion failure of the latter. The “non-filling of some specific items of the checklist” was a commonly reported reason for the non-completion. In several cases we identified the shortening or a combination of items, for example, demonstrating an adoption attempt even in critical situations. The WHO’s checklist is comprised of 19 items and WHO encourages the adaptation to each reality, though discouraging the exclusion of any item from the original proposition^1^. 

In addition to the discrepancy found between the number of complete and incomplete checklists, other limitations of the study are the retrospective data collection from an electronic database, being subject to information bias and to the absence of more specific clinical and laboratory data, and the realization in a single institution. Despite these limitations, the results are promising, providing evidence on the important contribution of the use of the surgical checklist in urgency situations in the reduction of surgical patients’ AEs, strengthening the premise that other studies should be carried out on this theme.

## CONCLUSION

The high rate of checklist completion in urgent colorectal surgeries verified demonstrated the feasibility of its use in urgency procedures. The higher rate of reoperation, readmission, and mortality in the small number of procedures with incomplete checklists denoted the impact of its use in the safety of patients undergoing urgent procedures. 

The most common reason for the non-completion of the checklist was the “absence of a team member”. The surgeon was the absent professional most of the times, indicating failure of teamwork in such situations, with possible repercussions on the lack of communication and, consequently, the occurrence of AE, and risking patient safety. 

The results of this study allow to infer that the use of the surgical checklist in urgent procedures is feasible and promising, and may reduce complications and mortality, thus promoting quality of care and patient safety.
